# Internal Factors Affecting the Crystallization of the Lipid System: Triacylglycerol Structure, Composition, and Minor Components

**DOI:** 10.3390/molecules29081847

**Published:** 2024-04-18

**Authors:** Dubing Yang, Yee-Ying Lee, Yuxia Lu, Yong Wang, Zhen Zhang

**Affiliations:** 1 JNU-UPM International Joint Laboratory on Plant Oil Processing and Safety, Department of Food Science and Engineering, Jinan University, Guangzhou 510632, China; 2 School of Science, Monash University Malaysia, Bandar Sunway 47500, Selangor, Malaysia; 3 Guangzhou Flavours & Fragrances Co., Ltd., Guangzhou 510632, China

**Keywords:** lipid crystallization, internal factor, minor component, molecular structure, physical property

## Abstract

The process of lipid crystallization influences the characteristics of lipid. By changing the chemical composition of the lipid system, the crystallization behavior could be controlled. This review elucidates the internal factors affecting lipid crystallization, including triacylglycerol (TAG) structure, TAG composition, and minor components. The influence of these factors on the TAG crystal polymorphic form, nanostructure, microstructure, and physical properties is discussed. The interplay of these factors collectively influences crystallization across various scales. Variations in fatty acid chain length, double bonds, and branching, along with their arrangement on the glycerol backbone, dictate molecular interactions within and between TAG molecules. High-melting-point TAG dominates crystallization, while liquid oil hinders the process but facilitates polymorphic transitions. Unique molecular interactions arise from specific TAG combinations, yielding molecular compounds with distinctive properties. Nanoscale crystallization is significantly impacted by liquid oil and minor components. The interaction between the TAG and minor components determines the influence of minor components on the crystallization process. In addition, future perspectives on better design and control of lipid crystallization are also presented.

## 1. Introduction

Lipids are widely used in food products. Lipid crystallization greatly influences the physical properties of lipids. The lipid crystal structure on multiple-length scales collectively affects the properties of lipids, such as the rheological characteristics, hardness, oil-binding capacity, and sensory attributes [1].

The crystallization process of lipids involves two steps: nucleation and crystal growth. However, crystallization can only occur after a driving force is applied. In a bulk lipid system, this driving force is often characterized by supercooling, which is the difference between the melting and crystallization temperatures. A higher degree of supercooling typically results in a faster crystallization rate [2]. The nucleation process can occur in various manners and is categorized into primary nucleation, which can be either homogeneous or heterogeneous, and secondary nucleation. Homogeneous nucleation takes place when the lipid system is devoid of any external particles and comprises solely crystallizing material. However, when there are external particles in the lipid system, heterogeneous nucleation could be induced through the interaction between the particle surface and crystallizing lipid. A lower driving force is needed for heterogeneous nucleation. Secondary nucleation is induced by seed crystal with the same crystallizing lipid [3]. Crystal growth occurs after the nucleation process, wherein growth units are incorporated into crystal nuclei. Three processes occur during crystal growth. Firstly, a TAG molecule is transferred to the crystal surface; subsequently, the molecule is incorporated into a kink site; and finally, latent heat is released [3]. The crystallization process dictates the crystal polymorphic form, nanostructure, microstructure, and therefore the physical properties of the lipid system.

Generally, there are three typical polymorphic forms of crystallized lipid: α, β’, and β, each with distinct subcellular structures and melting temperatures. Each polymorphic form has a different final Gibbs free energy, which in turn dictates the stability of the crystal structure. The α form exhibits significant instability owing to its greater Gibbs free energy, whereas the β’ form is more stable, and the β form has the highest stability. Therefore, upon the formation of the least stable α polymorph, it undergoes a polymorphic transformation into the β’ form, which then transitions into the most stable β form [4]. Due to the three-legged structure of a TAG molecule, two molecular conformations can be found in crystallized lipid: tuning fork and chair. The tuning fork configuration positions the *sn*-1 and *sn*-3 fatty acid chains in one direction while the *sn*-2 fatty acid chain faces the opposite direction. Conversely, in the chair configuration, the *sn*-2 fatty acid chain aligns with either the *sn*-1 or *sn*-3 fatty acid chain in one direction, while the other fatty acid chain faces the opposite direction. TAG molecules arrange themselves in pairs adjacent to each other within crystal planes, occasionally at varying angles. These TAG molecules can form either a double (2L) or triple (3L) chain-length structure [5].

Numerous investigations have been undertaken to clarify the effects of different external factors on lipid crystallization. Significant external factors encompass thermal processing, the use of shear, sonication, pressure, temperature, supersaturation, solvents, magnetic field, and electric field [4,6,7]. However, it is necessary to consider the internal factors. The lipid system is mainly composed of TAG, so the structure of TAG and the TAG composition determine the crystallization in the lipid system. Therefore, many investigations have been carried out to change the lipid composition through various modification processes, such as interesterification, fractionation, and hydrogenation. Minor components, especially additives, are usually classified as external factors. However, we defined minor components as internal factors since they are part of the lipid system. Minor components also play an important role, so some studies focus on the addition of minor components in order to modify lipid crystallization. One should note that the addition of minor components can alter the crystal forms rather than the polymorphism, as the chemical composition is changed by the minor components.

Following new insights into the hierarchical structure of lipid crystal, especially the nanostructure, many studies have explored how the structure of the nanocrystal is affected by various factors. These new insights have deepened our understanding of the formation of the crystal network. During the formation of the lipid crystal network, the initial steps involve the formation of nanocrystals, followed by the aggregation of these nanocrystals into clusters, finally leading to the establishment of a three-dimensional network [1]. Apart from the external factors, the effect of internal factors, such as TAG composition [8] and minor components [9,10] on lipid crystallization on the nano-length scale is also updated.

**Figure 1:**
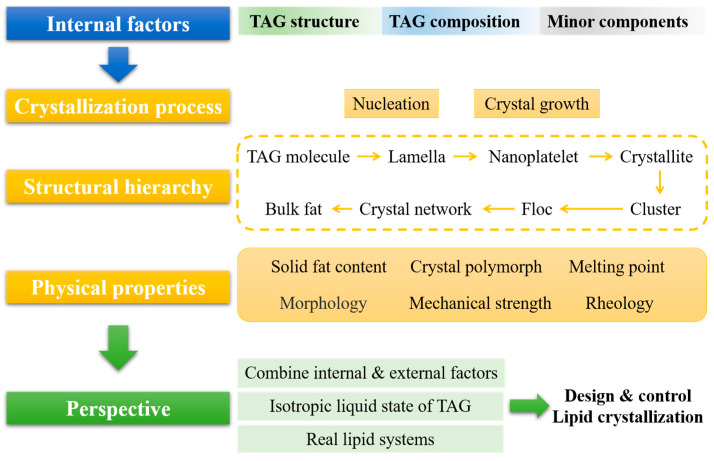
Schematic view for internal factors influencing different aspects of lipid crystallization.

The objective of this review is to offer a comprehensive summary of the internal factors, such as TAG structure (i.e., fatty acid structure and symmetrical/asymmetrical structures of TAG), TAG composition, and minor components, on lipid crystallization at different structural hierarchy levels and the resulting physical properties of the lipid system. Future perspectives on how various factors influence lipid crystallization, and thus on achieving a better design and control of lipid crystallization, are discussed (Figure 1).

## 2. Effect of TAG Structure

As the main component of the lipid system, TAG is a compound formed by combining glycerol with three fatty acids. These fatty acids can vary based on chain length, saturation level, and branching. The positioning of fatty acids along the glycerol backbone determines the stereospecific, symmetrical, or asymmetrical structure of TAG molecules.

Three key molecular interactions, including glycerol conformation, aliphatic chain packing, and methyl end stacking, significantly impact the polymorphic structures of TAG. These factors might interact in a complicated way [3]. TAG structure can affect these molecular interactions and thereby influence lipid crystallization. Similar characteristics among the three fatty acid chains result in the formation of 2L structures, while differences could increase steric hindrance, impeding their simultaneous arrangement within the same lamellar plane. The 3L structures emerge due to chain sorting [11].

### 2.1. Effect of Fatty Acid Structure

The effect of fatty acid chain structure on lipid crystallization is summarized in Table 1. Fatty acids typically possess a straight-chain structure. While they can vary in length, natural fatty acids commonly range from C4 to C22, with C18 being the most prevalent [12]. They are categorized based on carbon numbers into short-chain (≤6 carbons), medium-chain (8 to 14 carbons), and long-chain (≥16 carbons) fatty acids [12]. Various fatty acids influence distinct crystalline forms. In milk fat, TAG consisting of saturated long chain fatty acids forms the 2L crystal structure with a high melting point, while unsaturated long chain TAG contributes to the low-melting-point 3L structure. Additionally, asymmetrical TAG, featuring short chain fatty acids, exhibits a crystallization-inhibiting effect [13].

The longer the fatty acid chain length in TAG, the faster the nucleation process [14]. The fatty acid chain length also significantly affects the growth rate of TAG [5]. The linear crystal growth rates of monosaturated TAG decrease with increasing chain length, possibly because of the extended time needed for methyl chain ordering [5]. Longer fatty acid chains in TAG typically result in a higher melting point. Lipids with high melting points undergo a rapid crystallization process due to a greater crystallization driving force. However, it is also reported that lipids with longer chain saturated fatty acids in TAG resulted in an increased crystallization temperature, consequently decelerating the rate of crystallization [15]. The polymorphic behavior is also notably affected by the fatty acid chain length [5].

The evenness or oddness of the carbon number in the fatty acid chain influences the crystallization properties of lipids. The melting temperature tends to be lower for odd-numbered TAG compared to even-numbered TAG as the chain length increases. The impact is strongest with shorter chains and remains noticeable for the β polymorph with longer chains. This indicates a looser arrangement of crystals caused by the steric hindrance of the molecular structure in odd-numbered TAG, contrasted with the tighter packing observed in the β polymorph [5].

The differences in the fatty acid chain length of TAG are referred to as chain-length mismatch (CLM). CLM caused differences in the structure, crystallization, and melting behavior of 1,2-dipalmitoyl-3-stearoyl-*sn*-glycerol (PPS), 3-palmitoyl-1,2-distearoyl-*sn*-glycerol (PSS) and tristearoylglycerol (SSS) [16]. The symmetrical SSS, featuring a fairly even “terrace”, tends to crystallize most favorably in the highly stable β polymorph. The absence of two CH_2_ groups at the *sn*-1 position (PSS) causes sufficient structural disruption, encouraging the dominance and endurance of the β′ polymorph. However, the absence of four CH_2_ groups at both the *sn*-1 and *sn*-2 positions (PPS) creates a significantly greater disturbance, leading to a preference for the α polymorph [16]. CLM also influences the phase behavior of binary lipid systems. CLM exerts its influence mainly by changing the methyl end “terrace” through the interaction of CH_3_ groups from different layers. Increased CLM enables greater overlap of TAG chains at the methyl end, leading to the formation of a denser structure [17]. CLM affects the eutectic behavior and eutectic composition and the hardness of the binary systems [18].

Another factor is the presence of a double bond. The existence of a cis double bond in a fatty acid chain creates inflexible kinks, which disrupt the packing of the TAG molecules. TAG with double bonds exhibits lower melting points compared to saturated TAG [5]. A lipid rich in unsaturated fatty acids exhibits a lower melting point, resulting in decreased onset temperature of crystallization and lower nucleation rate [19]. Unsaturated fatty acids exhibit greater chain packing flexibility compared to saturated fatty acids. This increased flexibility reduces the activation energy needed for the transformation of unsaturated TAG into more stable forms [20]. In the isotropic liquid state, the presence of double bonds in triolein (OOO) makes the fatty acid chain more rigid, resulting in lower conformational conversion rates compared to SSS. This reduced flexibility is unfavorable for the crystallization process [21].

The distribution of double bonds within the fatty acid chain affects the crystallization. A pair of conjugated cis double bonds can lead to a straight alignment of the fatty acid chain, in contrast to two nonconjugated ones. Therefore, conjugated cis double bonds within TAG promote crystallization and lead to a higher melting point [22].

The presence of a trans double bond results in a straight fatty acid chain, which is more similar to a saturated fatty acid chain than a chain with a cis double bond. Therefore, the melting properties between TAG with trans double bond and its saturated counterpart are similar [22]. The presence of trans fat leads to much faster crystallization and affects the crystallization kinetics [23]. The effect of the trans double bond on lipid crystallization was significant when comparing trans TAG with both its cis counterparts and saturated counterparts [24]. 1,3-dipalmitoyl-2-elaidoyl-glycerol (PEP) and 1,3-dielaidoyl-2-palmitoyl-glycerol (EPE) stabilize in the 2L-β′ and 2L-β polymorphs, respectively. In contrast, the 3L-β polymorph is the most stable form for both 1,3-dipalmitoyl-2-oleoyl-glycerol (POP) and 1,3-dioleoyl-2-palmitoyl-glycerol (OPO), while the 2L-β′ form is the most stable for both 1,3-dipalmitoyl-2-stearoyl-glycerol (PSP) and 1,3-distearoyl-2-palmitoyl-glycerol (SPS) [24].

Branching in the chain reduces the melting point significantly [22]. Branched-chain fatty acid-TAG promoted the nucleation rate in the palm oil-based blend and contributed to the initial crystallization but retarded crystal growth, interfering with the tight packing of fatty acids. This leads to a less rigid, lower-melting-point structure [25]. High concentrations of branched-chain fatty acid-TAG exhibited a crystal dilution effect, which hindered the crystal growth and polymorphic transition from β′ to β, leading to a decrease in crystal density [25].

**Table 1:** Effect of fatty acid chain structure on lipid crystallization.

Fatty Acid Chain Structure	Lipid System	Effect			References
		Melting Point	Crystallization Process	Crystal Structure	
Fully saturated long chain	Milk fat	High	-	Double chain-length structure	[13]
Unsaturated long chain	Milk fat	Low	Inhibit	Triple chain-length structure	[13,19]
Short chain	Milk fat	-	Inhibit	Triple chain-length structure	[13]
Long fatty acid chain	Interesterified blends	-	Promote	-	[14]
Odd-numbered fatty acid chain	Pure TAG	Lower	-	Looser packing	[5]
Chain-length mismatch	Pure TAG	-	-	Less stable polymorphs	[16]
Chain-length mismatch	PPS/PSP, MMS/MSM, LLS/LSL, CCS/CSC binary systems	-	-	Denser structure	[17]
Cis double bond	Pure TAG	Lower	-	Looser packing	[5]
Conjugated cis double bonds	Pure TAG	Higher	Promote	-	[22]
Trans double bond	Pure TAG	Higher	-	-	[22]
Trans double bond	Palm-based confectionery fats	-	Promote	-	[23]
Branched fatty acid chain	Pure TAG	Lower	-	-	[22]
Branched fatty acid chain	Palm oil-based blend	Low	Promote nucleation; inhibit crystal growth	Looser packing	[25]

### 2.2. Effect of Symmetrical/Asymmetrical Structures of TAG Molecules

The structure of TAG is dictated not only by the structure of the fatty acids but also by the distribution of fatty acids in the glycerol backbone [12]. When a TAG molecule comprises only two types of fatty acids, its structure can be either symmetrical or asymmetrical, depending on the arrangement of the fatty acid chains. The stereochemistry of the glycerol backbone significantly influences the crystallization and polymorphic behavior of asymmetrical TAG. Despite similar melting points, pure enantiomers and their racemic blend exhibit distinct polymorphic tendencies. In many cases, the β′ form is the most stable polymorph for the pure TAG enantiomers, while the β form is the most stable polymorph for the racemic blend [26]. Craven and Lencki [26] demonstrated that in the β crystal structure, the unit cell comprises a pair of enantiomers of asymmetrical TAG, whereas in the β’ crystal structure, the unit cell consists of a single enantiomer. However, when TAG has an oleic acid located on the side of the glycerol backbone (*rac*-PPO, *rac*-SSO, *rac*-PSO, and *rac*-SPO), the TAG tends to form the β’ polymorph with 3L structure. In this structure, oleoyl chains are arranged in parallel between two leaflets consisting of saturated acyl chains. Mizobe et al. [27] reported the eutectic behavior in a blend consisting of 1-oleoyl-2,3-dipalmitoyl-*sn*-glycerol (S-OPP) and 1,2-dipalmitoyl-3-oleoyl-*sn*-glycerol (R-PPO) due to their different polymorphic behaviors compared to *rac*-PPO. Both S-OPP and R-PPO have 2L-α and 3L-β′ structures, while the racemic compound has 3L-α, 2L-β′, and 3L-β′ structures. β′-3 exhibits a higher melting point in both enantiomers than in their racemic mixture. The α form of the enantiomers crystallizes slower than the racemic compound. The chain-length structures were complex and distinct for the two enantiomers and the racemic mixture. Bayés-García et al. [28] investigated the effect of stereochemistry on the formation of molecular compound (MC) crystals. The POP/*sn*-PPO mixture and POP/*rac*-PPO mixture displayed different crystallization behaviors, with the POP/*sn*-PPO mixture showing separate crystallization.

Since the effect of stereochemistry on lipid crystallization has been discussed, the asymmetrical TAG is referred to as their racemic mixture in the following discussion. As summarized in Table 2, for pure TAG with both saturated and unsaturated fatty acid and TAG with different saturated fatty acids, symmetrical TAG showed a higher melting point and more polymorphic states than the unsymmetrical pair in their most stable form [29,30]. The crystallization behavior between symmetrical 1,3-distearoyl-2-oleoyl-*sn*-glycerol (SOS) and asymmetrical 1,2-distearoyl-3-oleoyl-*sn*-glycerol (SSO) is different. When oleic acid is located at the *sn*-1 or *sn*-3 position, the kink in the oleic acid causes a hindrance. Therefore, the end-group structure becomes unstable for SSO. In contrast, the end-group structure of SOS is more stable [31]. The polymorphic behavior depends on the symmetry of 1,2-dioleoyl-3-stearoyl-*sn*-glycerol (SOO) and 1,3-dioleoyl-2-stearoyl-*sn*-glycerol (OSO). In contrast to asymmetrical SOO, the cooling rate has a more profound impact on the symmetrical SOO. The asymmetrical structure of SOO causes extra steric hindrance and disruption in the “terrace” [32]. The symmetry of TAG also causes a difference in the crystallization of the α crystal. The crystallization of the α polymorph exhibits slower kinetics in POP than in PPO [33]. For a saturated asymmetrical/symmetrical TAG binary mixture, symmetrical TAG contributes to more stable crystal forms [34]. For a more complicated TAG mixture, asymmetrical TAG is generally β’-tending, while symmetrical TAG is β-tending [14]. In these systems, asymmetrical TAG delayed crystallization induction, led to a lower solid fat content (SFC), and modified the crystal microstructure [14]. Highly asymmetrical TAG could potentially hinder the transition from the α to β′ form during the crystallization process of milk fat [35] and inhibit the milk fat crystallization process [13].

## 3. Effect of TAG Composition

TAG is the main component in the lipid system, and its composition significantly influences crystallization properties. The crystallization and polymorphic properties of the lipid can be significantly influenced by even slight modifications in the TAG composition. Changes in TAG composition induce variations in molecular packing in the crystals, leading to alterations in polymorphism and crystalline domain size [37]. Generally, the similarity and variety of TAG in the lipid system are important aspects in terms of lipid crystallization. TAG molecules with similar structures compete for available positions within the crystal lattice, which slows down the crystallization kinetics in a multicomponent lipid system. Nevertheless, a specific combination of TAG can form a compound crystal, leading to the enhancement of crystal growth [12]. According to Larsson [38], the formation of β crystals is impeded by a more diverse range of fatty acids. The stability of a crystal structure typically increases with fewer types of TAG molecules present, as TAG molecules with similar chain lengths have a tendency to pack more tightly together [12]. An increase in SU_2_-TAG and S_2_U-TAG and diversity of fatty acid chain length was favorable for β′ formation, while β form was associated with S_3_ and U_3_, and TAG with a carbon number of 54 [14]. A higher TAG variety also results in a slow polymorphic transition [22] and a wide melting range due to complex phase behavior [39]. For a TAG mixture, the number of different TAG molecules determines the quantity of crystallized phases. Mixtures with k types of saturated TAG can exhibit (2k − 1) different β phases [40]. TAG composition could interact with other external factors such as shear, influencing the crystallization of a lipid [41].

### 3.1. Effect of High-Melting-Point TAG

**Figure 2:**
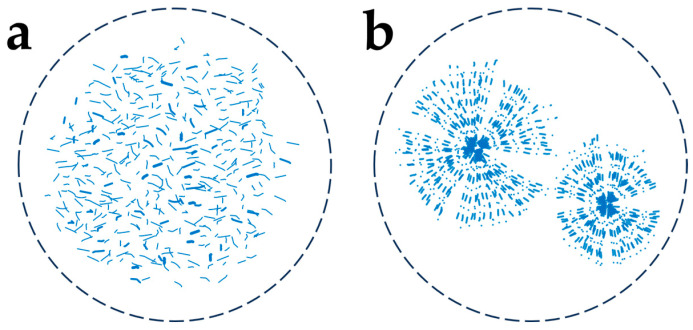
Graphical representation of crystal morphology of milk fat. (**a**) High concentration of high-melting-point TAG. (**b**) Low concentration of high-melting-point TAG.

TAG with a high melting point has a higher driving force for crystallization. Therefore, the crystallization of high-melting-point TAG occurs first and constitutes the crystal network skeleton [37]. TAG with a high melting point dominates thermal behavior in multicomponent lipid systems [42]. In the mixture of milk fat and fish oil, even a small incorporation of milk fat (high-melting-point TAG) could notably increase the crystallization and melting temperature of the mixture. The polymorphic behavior and crystal morphology were also dominated by milk fat characteristics [42]. The concentration of high-melting-point TAG determines the crystal growth mechanism in milk fat blends. The crystal growth pattern changed from multi-dimensional to one-dimensional with an increasing amount of high-melting-point TAG in the system. A high concentration of high-melting-point TAG resulted in small rod or needle-like crystals via one-dimensional growth, whereas a low concentration of high-melting-point TAG led to large spherical crystals through multi-dimensional growth (Figure 2) [43]. In a chemometrics study, the levels of saturated fatty acids and high-melting-point TAG were positively correlated with SFC. The higher the SFC, the higher the hardness and complex modulus [37]. Seilert et al. [44] investigated the model system containing long-chain and medium-chain saturated fatty acids and found that TAG containing three long-chain saturated fatty acids resulted in faster crystallization and polymorphic transformation kinetics. TAG with a high melting point also plays a key role in the formation of granular crystals in lipid blends. Excessive content of PPP and POP, known as high-melting-point TAG, hindered the growth of granular crystals [45]. The effect of TAG with a high melting point is obvious in the interesterified lipid systems. After the interesterification of palm olein, the formation of high-melting-point TAG shortened the induction time for crystallization, accelerated the crystallization process, and ultimately changed the morphology and texture [46]. The addition of high-melting-point TAG reduced the time required for nucleation to begin, resulting in increased firmness and heat resistance [47]. Therefore, high-melting-point hard fats can serve to modify and facilitate the crystallization process as seeding agents [48]. This is further discussed in Section 4.2.

### 3.2. Effect of Liquid Oil

The most efficient and cost-effective approach to altering the crystallization behavior and structural characteristics of lipids is through physical blending, which involves using liquid vegetable oils to adjust the lipid composition [8]. A high content of liquid oil typically contains a low amount of SFA, leading to the formation of a crystal with a soft texture. This increases the risk of structural loss during storage, and some oil might eventually separate from the crystal [49]. The crystallization behavior, crystal structure, and physical characteristics change with the inclusion of liquid oil [8]. Liquid oil delays the nucleation and crystal growth process [8], but promotes the polymorphic transition by creating more room for conformational changes in TAG within the crystal network [50]. However, a higher proportion of olein was found to be responsible for delaying the polymorphic transformation from the α form to the β′ form in milk fat [51]. An increased amount of liquid oil resulted in a decrease in the melting temperature and SFC due to higher TAG solubility [52]. Liquid oil could increase the size of the crystal domain with more liquid oil between the lamellae. This resulted in a crystal network with larger crystals and a smaller fractal dimension, which further weakened the interaction among the crystal clusters. As a result, the crystal network exhibited a softer texture [8]. Liquid oil might also contribute to the formation of granular crystals. The migration of crystals could be enhanced by the presence of liquid oil in the system, thereby facilitating the formation of granular crystals [53]. The dilution effects of liquid oil can result in fat bloom and softening during temperature fluctuations in chocolates [54]. The compatibility of the lipid blend could be improved by adding liquid oil into the system [55]. Pellegrino et al. [56] illustrated the impact of OOO on phase behavior in OOO/PPP/SSS ternary blends. At low OOO concentration, the system exhibited phase separation, with incorporation of OOO into SSS and PPP crystals. When increasing the OOO concentration to an intermediate level, the lipid system consisted of a PPP/SSS solid solution and OOO due to the poor accommodation of liquid OOO within the PPP/SSS. At a high concentration of OOO, the blend consisted of co-crystals of PPP/SSS dispersed in a liquid phase of OOO.

### 3.3. Effect of Specific TAG Combination

Because lipid systems often contain multiple types of TAG, the crystallization and physical properties are influenced by the combined effects of these TAG molecules [11]. The molecular interaction in TAG combinations can cause three types of phase behavior: miscible, eutectic, and molecular compound [57]. Macridachis-González, Bayés-García and Calvet [11] have reviewed the solid-state miscibility of binary and ternary systems. The miscibility of TAG molecules that are fully saturated with a single type of acid and those that have a mixture of saturated and unsaturated acids depends on the methyl-end plane configuration, with less tightly packed methyl-end planes favoring miscibility in metastable forms. In TAG with both saturated and unsaturated fatty acid, the positioning of adjacent glycerol groups and the steric hindrance of different acyl chains determine the formation of immiscible phases or MC in binary TAG mixtures [11]. Introducing a third TAG into a binary TAG system significantly affects the miscibility properties of the entire system [11].

Recent research has concentrated on the formation of MC in lipid systems. Generally, MC in TAG systems results from a 1:1 ratio of saturated–unsaturated mixed-acid TAG components, organized in a well-defined manner within the crystal lattice. This stoichiometric compound displays distinctive structural and thermodynamic properties attributed to the specific molecular interactions among individual TAG molecules [57]. The formation of MC crystals is promoted by three key factors: conformational stabilization of the glycerol group, π–π interactions between the unsaturated fatty acid chains, and the stable arrangement of saturated and unsaturated chains [57]. MC also exhibited unique properties in interaction with a third component, particularly with high-melting-point components. MC crystals containing cocoa butter and symmetrical/asymmetrical stearic-oleic mixed-acid TAG exhibit a 2L-β polymorphic structure [58]. PPP and MC_POP/OPO_ crystallize and undergo polymorphic transitions independently in PPP/MC_POP/OPO_ equimolecular mixtures. These behaviors were influenced by differences in the heat resistance of TAG and in steric hindrance arising from interactions between saturated and unsaturated fatty acid chains. MC_POP/OPO_ was able to adjust the thermal properties by integrating itself into the β phase of PPP [59]. In PPP/MC_POP/PPO_ mixtures, the monounsaturated TAGs showed similar incorporation into the solid solution with PPP, and MC crystals of POP and PPO were formed in eutectic compositions [60]. Eutectic TAG mixtures containing MC crystals exhibit interactive polymorphic crystallization behavior. The β crystal of MC_SOS/OSO_ promoted the polymorphic transformation of trilaurin (LLL) from the β′ to the β form, which is also termed “polymorphic stabilization”, by the mechanisms of melt-mediated transformation or “epitaxial effects” [61].

Besides the composition of TAG, specific interaction among TAG molecules also influences the physical properties and polymorphic structure of cocoa butter [62]. In cocoa butter, the polymorphic transition rate from the IV to V crystal is controlled by the interactions of POS/POP and POS/SOS. The IV structure of POS/SOS is templated by these interactions [63]. In the POP, POS, and SOS ternary mixtures, POP exhibited eutectic behavior with both POS and SOS, which reduced the melting point of the system [64]. The phase behavior of the SSO/SSS TAG mixture was investigated by Wijarnprecha et al. [65]. SSO acted as a solvent for SSS, thereby reducing the crystallization and melting temperatures of the mixture. SSO could facilitate the formation of the β′ crystal of SSS by integrating into the solid state, while a small amount of SSS could also promote the formation of the 3L-β′ crystal of SSO.

### 3.4. Effect of Main TAG Ratio

When a lipid system mainly consists of TAG with similar properties (e.g., melting point), the ratio of these main TAGs also influences crystallization. Cocoa butter is mainly composed of POP, SOS and POS, the ratio of which affect the polymorphism, crystallization kinetics, and polymorphic transformation. There are more polymorphic forms in SOS-rich cocoa butter equivalent (CBE) than in POP-rich CBE and cocoa butter. A high amount of POP delayed the polymorphic transition into the 3L-β crystal [66]. In another study of CBE, the impact of the ratio of POP to POS was significant. Isothermal crystallization at 20 °C accelerated with an increase in POS levels. A higher amount of POP decelerated the crystallization process in CBE but resulted in a minor formation of the V crystal transitioning from the II crystal [67]. Polymorphic phase behavior depends on the ratio of the main TAG. For the binary LLL/trimyristin (MMM) mixtures, rapid cooling caused the formation of two immiscible α phases, both consisting of LLL and MMM with varying proportions. Increasing the amount of MMM also leads to the expansion of the interlamellar spacing of the α phases. An optimal ratio of approximately 4:1 for the LLL and MMM molecules was identified for effectively incorporating the larger MMM into an α phase with LLL predominance. In addition, there were three different β phases in the LLL/MMM TAG systems: pure β_LLL_ phase, mixed β_LLL/MMM_ phase, and pure β_MMM_ phase. Understandably, the LLL/MMM ratio determined the proportions of these phases, with the intermediate ratio favoring the formation of mixed crystals of LLL/MMM. However, regardless of the main TAG ratio in the mixture, pure β phases always coexisted with a mixed β phase [40]. Vereecken et al. [68] investigated model fat blends containing identical levels of saturated fat but varying ratios of symmetrical and asymmetrical monounsaturated TAG. In stearic-rich blends, the asymmetrical blend exhibited slower crystallization kinetics, which was attributed to fractional crystallization. In contrast, a high ratio of symmetrical TAG showed faster crystallization behavior [69], leading to a denser crystal network [70]. The texture of fat blends with a high ratio of symmetrical monounsaturated TAG was more sensitive to shear than that of fat blends with a low ratio of symmetrical monounsaturated TAG [41].

## 4. Effect of Minor Components

**Figure 3:**
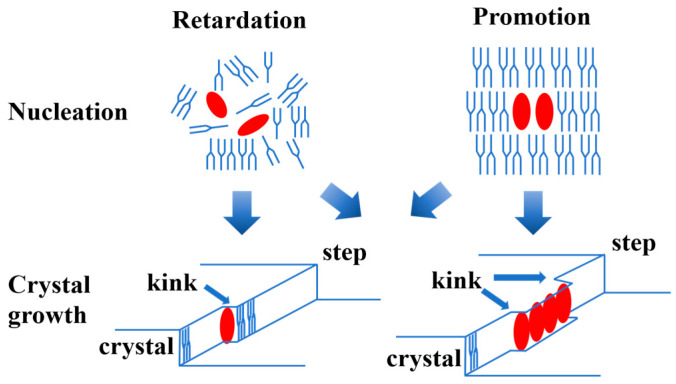
General effects of minor components on nucleation and crystal growth.

The influence of minor components on various aspects of crystallization cannot be underestimated. The general effects of minor components on nucleation and crystal growth are depicted in Figure 3. Nucleation is hindered if the minor component disrupts crystal nucleus formation, but promoted if it acts as a template. Crystal growth is impeded if the minor components hinder the attachment of TAG molecules at the kink sites, yet facilitated if they create new kink sites at the molecular step [3]. The interaction between minor components and the bulk lipid determines the influence of minor components on lipid crystallization [10]. Additionally, the path of lipid crystallization and the resulting physical properties, such as spreadability and hardness, depend on the interaction of these components [71]. Despite extensive research on this topic, ongoing studies continue due to the diverse materials influencing this process and the increasing interest in the nanostructure of lipid crystals.

### 4.1. Effect of Native Minor Components

Monoacylglycerols (MAGs), diacylglycerols (DAGs), fatty acids, and phospholipids constitute the main native minor components in natural lipids [3]. The systematic evaluation of how native minor components affect lipid crystallization has been conducted based on the hierarchical structure of the lipid crystal network [10,72]. In coconut oil and palm kernel oil, it was found that native minor components are responsible for the formation of the initial crystals. The crystallization induction time decreased, and the nucleation temperature increased due to the combined effect of the native minor components and high-melting-point TAG. The rate of crystal growth significantly slowed down because native minor components were absorbed at the kink sites, obstructing other TAG molecules from incorporating into the crystal structure. The native minor components did not affect the crystal morphology transformation, but removing them increased the thickness of nanoscale crystals, leading to a change in the lipid crystal network. As a result, the microstructure networks transitioned from a less-refined crystal structure to a more-refined one [72]. Similar results were found in fully hydrogenated coconut oil and fully hydrogenated palm kernel oil. Moreover, the minor components were related to the formation of less-stable polymorphs [10]. DAG is significant in research on fat crystallization because of its abundance within the lipid system. Due to the similarity in fatty acid composition between native DAG and the bulk lipid, native DAG is expected to exert a stronger impact on lipid crystallization compared to non-native DAG [3]. The native DAG enhanced TAG nucleation while impeding crystal growth, altering crystallization kinetics at various temperatures. DAG molecules attached to kink sites on the crystal–liquid interface, hindering lamellae incorporation and modifying the lipid crystal into a more unstable form. This led to thinner crystalline domains, observed as larger nanoplatelets with a smoother surface in 2%-DAG fat. Notably, DAG induced significant microstructural differences, featuring smaller crystals and a higher fractal dimension at elevated crystallization temperatures [9]. The impact of DAG consistently aligns with that of all native minor components, as reported by Chai, Meng, Cao, Liang, Piatko, Campbell, Lo and Liu [10], indicating the determining effect of DAG. The presence of free fatty acids did not modify the nanostructure of anhydrous milk fat; however, it altered the microstructure and modified the texture properties of the milk fat. This suggests that there is no direct connection between the nanostructures and the microstructures [73].

### 4.2. Effect of Seeding Agents

The relationship between the seeding agents and bulk lipid determines the effect of the seeding agents on lipid crystallization, as revealed through three important factors: the degree of supercooling, the compatibility in structure, and the polymorphic matching [74]. The seeding agents have a higher melting point than the bulk lipid; therefore, they experience a greater degree of supercooling within the lipid system and nucleate rapidly, thereby inducing heterogeneous nucleation within the lipid matrix. Moreover, seeding agents have a greater effect on lipids that exhibit a lower degree of supercooling [74]. Improved structural compatibility between seeding agents and bulk lipid typically encourages the development of larger crystals through epitaxial growth [74]. Structural compatibility is generally determined by the difference in chain length between seeding agents and TAG in bulk lipid. For seeding agents (PPP and SSS) with longer chain lengths than the bulk lipid (coconut oil), heterogeneous nucleation is the effect of seeding agents on bulk-lipid nucleation. Moreover, the magnitude of the difference in chain length between seeding agents and TAG in bulk lipid also significantly influences the crystallization of bulk lipid. With the addition of 1 wt% SSS seeding agent, coconut oil directly crystallized into the β′ crystal, and the crystallization of unstable crystal phases was inhibited. In contrast, the use of 1 wt% PPP did not alter the polymorph of coconut oil, but rather promoted the crystallization of the unstable phases [75]. Moreover, the effect of these seeding agents also depends on concentration and cooling rate [75]. The effect of polymorphic matching between seeding agents and coconut oil was further investigated by Mahisanunt et al. [76]. With the addition of seeding agents at 1 wt%, the nucleation of coconut oil was induced by the polymorphic matching between the α and β’ crystals of the seeding agents and the β’ crystal of coconut oil through epitaxial growth, with the β’ form showing a more pronounced effect. In contrast, the crystallization of long-chain TAG in coconut oil was promoted by the β crystal of the seeding agents through a templating effect [76]. In another study, the transition from β’ form to β form was delayed by the addition of α form monobehenin (1 wt%) to palm stearin due to polymorphic mismatching [74]. The effect of seeding agents is also determined by the combination of chain-length difference and polymorphic matching. When adding 0.5–3.0 wt% seeding agents, 1,3-dipalmitoyl-2-behenoyl-glycerol (PBP) (3L-β tending) promoted the nucleation of palm stearin by ordinary heterogeneous nucleation, while 1,3-dipalmitoyl-2-stearoyl-glycerol (PSP) (2L-β′ tending) promoted the nucleation by epitaxial growth. This is due to the small difference in chain length and better polymorphic matching between PSP and the main TAG in the bulk lipid [77]. Co et al. [78] investigated the effect of SSS (1–4 wt%) on the crystallization of 1,3-Distearoyl-2-oleoylglycerol (SOS) using computer simulations. SSS promoted the heterogeneous nucleation of SOS by forming “planar methyl surfaces” for the adsorption of SOS. The addition of SSS decreased the surface free energy, activation free energy, and critical radius of the nucleus, thereby increasing the nucleation rate [78].

### 4.3. Effect of Organic Additives

Table 3 summarizes the effect of various organic additives on lipid crystallization, based on the recent literature. Emulsifiers, also referred to as surfactants or surface-active agents, are a category of substances possessing both hydrophilic and lipophilic groups [79]. Emulsifiers are the primary organic additives used to modify lipid crystallization, such as nucleation, crystal growth, and polymorphic transformation processes. The impact of emulsifiers on crystallization is influenced by several factors, including the nature of their polar head groups and fatty acid chains, their hydrophobic or hydrophilic characteristics, concentration, solubility within the bulk lipid, similarity in chain length between emulsifiers and the bulk lipid, cooling rate, and the polymorphic form of the lipid [3]. It is worth noting that the effect of emulsifiers is based on their molecular structure rather than their surface activity, and hydrogen bonds can be formed between the carbonyl groups of TAG and the hydrophilic group of emulsifiers [80].

The ability of waxes to alter lipid crystallization stems from the long fatty acid and alcohol chains. Natural waxes were reported to promote crystallization, change the crystal growth mode and introduce a new hydrocarbon chain, thus changing the crystal morphology [102]. Natural waxes could increase firmness, possibly due to their role in forming the structural foundation of the crystal [103]. The chain length of waxes affects the interactions of waxes and TAG molecules. Waxes with relatively shorter chain lengths served as seeding agents to promote epitaxial growth in a mixture of cocoa butter and coconut oil eutectics, while waxes with longer chains acted as nucleation sites to encourage heterogeneous nucleation [104]. The chain length of the waxes also affects the lamellar thickness and nanocrystal size [103].

Sugar is often incorporated into a continuous lipid matrix. The addition of sugar typically facilitates lipid crystallization, inhibits the formation of the β polymorph, strengthens the crystal network, and enhances storage modulus and firmness by promoting interfacial interactions [99]. The SFC of palm oils was reduced by the addition of sugar, while their solid-like characteristics were enhanced, which is attributed to the influence of sugar dispersion on oil viscosity [111]. The effect of sugar also depends on particle-size distributions. Small sugar particles serve as junction points, increasing the connectivity between sugar and bulk lipid [98]. Particle interaction increases with smaller sugar particles and a higher sugar proportion in the chocolate system, resulting in lipid migration and recrystallization [112]. Moreover, the diverse shapes and properties of sugar particles lead to varying effects on the formation of chocolate bloom. During storage, fat bloom was encouraged by sucrose, whereas chocolate systems containing maltitol, corn-syrup solids, and polydextrose particles did not display notable bloom [113]. In addition, the presence of sugar crystals restrains the impact of emulsifiers on the crystallization of cocoa butter and retards its transition from the IV form to the V form [100].

Limonene, a small hydrophobic molecule, has been deemed a potential modulator of lipid crystallization. The liquid state of limonene resulted in a dilution effect in the lipid system, where it acted as a physical barrier, disrupting the packing of TAG and consequently decreasing SFC [109]. Therefore, limonene can reduce SFC and delay crystallization and accelerate polymorphic transition [107,108,109].

### 4.4. Effect of Inorganic Additives

Inorganic additives, despite their lack of acyl groups in their chemical structure, demonstrate a notable potential to promote crystallization in lipid systems. Yoshikawa et al. [114] investigated the influence of inorganic additives on the crystallization of LLL. The additives enhanced crystallization and facilitated the transformation from the β’ form to the β form. TAG molecules were adsorbed onto talc and graphite surfaces in different orientations. The talc surface was parallel to the lamellar planes of LLL, whereas the graphite surface was perpendicular to the lamellar planes [114]. Talc nanoparticles showed heterogeneous nucleation effects in cocoa butter. Talc also significantly increased the crystallization temperatures of cocoa butter and promoted the formation of more stable polymorphic forms. Moreover, the concentration of talc determined the orientation of the TAG molecules. A concentration of 0.1% talc led to a highly ordered orientation, with the lamellar planes parallel to the talc surface. However, when increasing the talc concentration to 0.5%, the ordered orientation was disrupted due to the randomly dispersed talc particles throughout the cocoa butter system [115]. The effect of the graphite was further associated with its surface, which featured a hexagonal network of carbon. This surface promoted heterogeneous nucleation, thereby facilitating the formation a of 2L-β crystal [116]. The effect of dispersed particles on lipid crystallization also depends on particle surface chemistry. During static crystallization, silica (with a hydrophilic surface) tended to be more effective in reducing the proportion of 2L-β’ PPP crystal than octadecyl-functionalized silica (with a hydrophobic surface). Silica was better at enhancing 2L-α POP crystal formation upon the application of shear [117]. Moreover, dissolved carbon dioxide (CO_2_) was found to influence lipid crystallization. Truong et al. [118] reported that dissolved CO_2_ promoted the nucleation and crystal growth of anhydrous milk fat. CO_2_ also induced the α form crystal formation and led to a decrease in the size of the crystal and an increase in the overall quantity of crystal. The presence of CO2 altered the thermal characteristics and texture. CO_2_ was found to increase SFC and the hardness of the anhydrous milk fat [119]. CO_2_ was also found to amplify the effects of sonication in promoting the crystallization of anhydrous milk fat [120].

## 5. Discussion and Perspectives

### 5.1. Combined Effect of Internal and External Factors

Apart from the internal factors mentioned above, the crystallization of lipids is also significantly influenced by the external factors. External factors can alter lipid crystallization kinetics and ultimately change the physical properties of lipid crystals, such as texture and melting point [121]. These external factors include thermal treatment, shear, sonication, pressure, magnetic field, and electric field. Internal factors might make lipid crystallization more sensitive to external factors [99]. External factors might even dominate over internal factors [95]. In some cases, there is a strong interaction effect between internal factors and external factors [122]. Hence, it is recommended that forthcoming studies place emphasis on examining the interaction between internal and external factors in the context of lipid crystallization.

### 5.2. Isotropic Liquid State

While most studies focus on the final state of crystallization, the exploration of TAG arrangements in the liquid state remains limited. The molecular arrangement of TAG in the liquid state has a major impact on nucleation and crystal formation [21]. There could be four different molecular conformations in the liquid state (i.e., trident, chair, propeller, and tuning fork). However, there is ongoing debate on whether TAG molecules are randomly oriented or exhibit molecular organization at temperatures well above their melting point [123]. Golodnizky and Davidovich-Pinhas [124] proposed a “supply and demand” theory for tuning-fork TAG to relate the liquid state to the solid state. In a following study, Golodnizky et al. [125] identified a connection between the isotropic liquid phase and the crystallization mechanism of cocoa butter, highlighting the significance of POS in cocoa butter crystallization. How the TAG structure and composition affect the arrangement in the liquid state is an unanswered question.

### 5.3. Real Lipid Systems

Fundamental studies have extensively investigated the crystallization mechanism in pure TAG or their mixture systems over the years. However, understanding how internal factors affect the crystallization mechanism in real lipid systems is still a challenge. The knowledge from fundamental studies might not be straightforwardly applied to real lipid systems due to the complicated composition of these systems. For example, the mechanism of MC formation in TAG mixtures remains unclear [123]. This challenge could be addressed through the following aspects. A practical way to study the crystallization mechanism in real lipid systems is to analyze the crystallized fractions at different stages of crystallization by separating crystals from the liquid fraction. Another issue that needs to be addressed is the accurate identification of the TAG composition, especially the precise analysis of TAG regioisomers. The accurate analysis of TAG composition is instrumental in relating the TAG composition to the crystallization. In addition to direct experimental methods, molecular dynamics simulations could be a promising method for studying the crystallization mechanism in real lipid systems, even though it is still at an early stage. The challenge in conducting molecular dynamics simulations lies in the large number of atoms contained within lipid molecules [123]. However, with the rapid development of computational power, it is expected that more insights into real lipid systems will be gained through this method.

## 6. Conclusions

This review summarizes the internal factors influencing lipid crystallization, encompassing TAG structure, TAG composition, and minor components in the lipid system. The effects of these factors interact with one another, constituting an overall internal influence on the lipid crystallization process across multiple length scales and thereby imparting specific physical properties to the lipid system. Variations in fatty acid chain length, double bonds, and branching, along with their arrangement on the glycerol backbone, dictate molecular interactions of TAG. High-melting-point TAG dominates the crystallization process, while liquid oil hinders this process but facilitates polymorphic transitions. Unique molecular interactions arise from specific combinations of TAG, yielding MC crystal with distinctive properties. The presence of liquid oil and minor components significantly impacts nanoscale crystallization. The impact of minor components on the crystallization process is determined by the interaction with the bulk lipid. By further understanding various factors influencing lipid crystallization, we can enhance our ability to design and control the crystallization of lipid systems, achieving the targeted physical properties by manipulating the composition of the lipid system and selecting appropriate external conditions.

## Figures and Tables

**Table 2:** Thermodynamic parameters of homologous TAG pairs *.

Homologous TAG Pairs	Tm (°C)			ΔH (kJ/mol)			References
	Symmetrical	Asymmetrical	1:1 Mixture	Symmetrical	Asymmetrical	1:1 Mixture	
POP/PPO	36.6	34.6	32.4	144	104	110	[36]
SOS/SSO	43.8	42.4	40.6	159	124	124	[36]
OPO/POO	21.0	19.5					[29]
OSO/SOO	25.4	24.5					[29]
PSP/PPS	70.8	59.2	62.4	130	113	124	[30]
CSC/CCS	43.5	30.4	23.0	156	128	123	[17]
LSL/LLS	48.8	38.2	42.6	105	98	105	[34]

* ΔH, the molar heat of fusion ΔH; Tm, melting point; P, palmitoyl; S, stearoyl; O, oleoyl; L, lauroyl; C, caproyl.

**Table 3:** Effect of organic additives on lipid crystallization.

Additive	Additive Amount	Bulk Lipid	Effect	References
Glyceryl monostearate	1–4 wt%	Palm stearin	Glyceryl monostearate increased the crystallization temperature and induced isothermal crystallization. Glyceryl monostearate promoted polymorphic transformation from the α form to the β′ form. An amount of 4% of glyceryl monostearate reduced the crystal size.	[81]
Sorbitan monopalmitate, glyceryl monostearate, and glycerol monopalmitate	4%	Palm oil	Sorbitan monopalmitate, glyceryl monostearate, and glycerol monopalmitate significantly improved the formation of β crystals. Glyceryl monostearate promoted crystal growth by the absorption of molten TAG molecules during recrystallization.	[82]
Polyglycerol ester of fatty acids (PGE)	0.5–5 wt%	Palm olein	PGEs decreased solid fat content, decreased the number of crystals, and increased the crystal size. PGE1105 and PGE1117 promoted early crystallization but hindered later stages, possibly through template effects, while PGE1155 retarded the entire crystallization process.	[83]
Sorbitan monopalmitate (SM)	1–5 wt%	Mango butter	SM promoted the aggregation of globular mango butter crystals. SM disrupted the crystal structure of mango butter.	[84]
Polyglycerol polyricinoleate (PGPR)	1–10 wt%	Mango butter	PGPR modified the microstructure of mango butter. PGPR caused imperfections in the mango butter crystal. PGPR changed the rate of nucleation and crystallization. PGPR decreased the mechanical strength.	[85]
Sucrose esters	1 wt%	Cupuassu fat	Sucrose esters promoted nucleation and increased the crystallization rate. Sucrose esters favored the formation of β′ form crystal during thermal cycling. Sucrose esters promoted the formation of β crystal in storage condition.	[86]
Sucrose esters (S170, P170 and L195)	0.5–5 wt%	Palm olein	S170 and P170 promoted early crystallization through template effects but inhibited crystallization in later stages. L195 inhibited crystallization due to structural differences.	[87]
Sucrose behenate	1%	Soft fats	Sucrose behenate improved the thermal stability and altered the hardness. Sucrose behenate significantly modified the crystal network structure. Sucrose behenate retarded the polymorphic transition from α form to β form.	[88]
Hydrophobic sucrose esters	0.4 wt%	Hydrogenated palm kernel oils	Sucrose esters with low melting points caused the formation of bigger and rougher fat crystals. Sucrose esters with low melting points induced nucleation and caused smaller and more-uniform crystal.	[89]
Sorbitan tristearate and sucrose stearate	1–5 wt%	Palm mid-fraction	Sorbitan tristearate increased the liquid fraction of PMF and led to liquid-mediated transformation. Sucrose stearate delayed the α crystal formation.	[80]
Sorbitan tristearate and sucrose stearate	1–5 wt%	Palm oil	Sorbitan tristearate and sucrose stearate changed the microstructure and increased the hardness. Sucrose stearate mainly influenced high-melting-point TAG crystallization, while sorbitan tristearate affected both high- and low-melting-point TAG crystallization. Sorbitan tristearate improved the polymorphic stability, while sucrose stearate softened the texture.	[90]
Sucrose stearate and sucrose behenate	0.1–0.5 wt%	Cocoa butter	Sucrose stearate and sucrose behenate promoted faster crystallization. Sucrose stearate and sucrose behenate modified the solid dissolution process and oil migration through cocoa butter and altered physical properties.	[91]
Polyglycerol ester	0.05–0.25 wt%	Anhydrous milk-fat and hydrogenated palm-kernel-oil blend	Polyglycerol ester promoted the nucleation. Polyglycerol ester caused the formation of small and uniform crystals.	[92]
Span-60, sucrose ester S-170, Span-80), and sucrose ester O-170	0.1 wt%	Anhydrous milk-fat and hydrogenated palm-kernel-oil blend	Span-60 and S-170 resulted in tiny and uniform crystals. Span-80 and O-170 caused loose and large crystals.	[93]
Sorbitan tristearate and sorbitan tribehenate	5 wt%	Palm mid-fraction	Sorbitan tristearate promoted the formation of α crystals. Sorbitan tribehenate induced heterogeneous nucleation and accelerated crystallization in the β′ form.	[94]
Lecithin	1%	Palm oil	Sunflower lecithin stabilized the β′-form crystal.	[95]
Phospholipids	0.3–0.8 wt%	Cocoa butter	Phospholipids significantly increased crystallization rate and extent in model chocolates. Phospholipids can improve the microstructural stability, reducing fat migration and preventing bloom formation.	[96]
L-ascorbyl palmitate	1–5 wt%	Palm oil	L-ascorbyl palmitate accelerated the isothermal crystallization. L-ascorbyl palmitate promoted the transition from β crystal to β′ crystal. L-ascorbyl palmitate reduced the thickness of the nanocrystal. L-ascorbyl palmitate led to small and uniform crystals.	[97]
Sugar	5–50 wt%	Palm oil	Sucrose increased the crystallization rate. Sucrose served as a “bound filler”. Small particles significantly enhanced the elasticity characteristics. Small particles enhanced viscoelastic properties to the same extent as those of high-melting-point fats.	[98]
Sugar	50 wt%	Palm-oil and mid-fraction blend	Sugar increased the sensitivity of fat to processing conditions. Sugar increased the hardness and elasticity of the crystal network. Sugar inhibited the formation of β crystals.	[99]
Sugar and emulsifiers	2 wt% emulsifier 50 wt% sugar	Cocoa butter	Emulsifiers with low molecular weight accelerated crystallization. Sugar accelerated crystallization and suppressed the transformation from IV to V. Sugar negated the impact of emulsifiers on crystallization.	[100]
Alternative sweetener and carbohydrate polymer mixtures	46 wt%	Cocoa butter	Alternative sweetener promoted the crystal packing and led to a firmer texture. The tiny particle size prompted the unstable γ polymorph of TAG to crystallize into a more stable form.	[101]
Beeswax and carnauba wax	2–8 wt%	Palm kernel stearin	Carnauba wax accelerated crystallization. Beeswax and carnauba wax introduced new hydrocarbon chain distances. Beeswax and carnauba wax reduced the nanocrystal size and lamellar distance. Beeswax and carnauba wax altered the crystal morphology.	[102]
Candelilla wax and rice bran wax	2–8 wt%	Palm kernel stearin	Candelilla wax promoted the crystallization process. Candelilla wax and rice bran wax introduced new hydrocarbon chain distances. Rice bran wax increased the thickness of lamellar and nanocrystal size. Candelilla wax led to small uniform crystal, whereas rice bran wax led to large rod-like layered crystal. Candelilla wax and rice bran wax resulted in higher firmness.	[103]
Derivatives of paraffin waxes (N-alkanes)	1–5 wt%	Cocoa-butter and coconut-oil blend	N-alkanes induced heterogeneous nucleation and promoted the crystallization of the blend. N-alkanes interacted more dominantly with coconut oil than with cocoa butter	[104]
Rice bran wax	1–5 wt%	Cocoa butter	Rice bran wax accelerated the tempering process and V-crystal formation. Rice bran wax delayed the transition from V to VI. Rice bran wax delayed the formation of fat bloom during storage.	[105]
Essential oils (5% *w*/*w*) obtained from the flowers (EsOF) and stems (EsOS) of *Pituranthos scoparius*	5 wt%	Palm oil-based fats	Essential oils decreased the rate of crystallization and SFC. EsOF led to a less organized crystal network, whereas EsOS led to more organized crystal network. EsOF resulted in bigger crystals in palm oil while EsOS led to smaller crystals.	[106]
Limonene	1–10 wt%	Palm oil	Limonene decreased the SFC and consistency. Limonene can alleviate the post-hardening phenomenon.	[107]
Limonene	1–10 wt%	Palm stearin	Limonene promoted crystallization. A high concentration of limonene reduced the crystal size and accelerated the polymorphic transformation to the β crystal.	[108]
Limonene	1–10 wt%	Palm olein	Limonene alleviated clouding. Limonene reduced crystallization temperature and cloud point. Limonene inhibited the nucleation of the high-melting-point TAG. Limonene inhibited crystal growth and agglomeration.	[109]
Cannabidiol	1–2.5 wt%	Anhydrous milk fat, palm oil, palm kernel oil, and cocoa butter	Cannabidiol delayed the crystallization of all fats. Cannabidiol slightly increased the crystal size for all lipid samples. Cannabidiol increased hardness and elasticity. Cannabidiol had different effects on different lipids.	[110]

## Data Availability

The data presented in this study are available on request from the corresponding author.
